# A Dictionary Learning Approach with Overlap for the Low Dose Computed Tomography Reconstruction and Its Vectorial Application to Differential Phase Tomography

**DOI:** 10.1371/journal.pone.0114325

**Published:** 2014-12-22

**Authors:** Alessandro Mirone, Emmanuel Brun, Paola Coan

**Affiliations:** 1 European Synchrotron Radiation Facility, BP 220, F-38043, Grenoble, France; 2 Ludwig Maximilians University, Physics Department, 85748, Garching, Germany; 3 Institute for Clinical Radiology, Ludwig-Maximilians-University Hospital Munich, Munich, Germany; University of Pécs Medical School, Hungary

## Abstract

X-ray based Phase-Contrast Imaging (PCI) techniques have been demonstrated to enhance the visualization of soft tissues in comparison to conventional imaging methods. Nevertheless the delivered dose as reported in the literature of biomedical PCI applications often equals or exceeds the limits prescribed in clinical diagnostics. The optimization of new computed tomography strategies which include the development and implementation of advanced image reconstruction procedures is thus a key aspect. In this scenario, we implemented a dictionary learning method with a new form of convex functional. This functional contains in addition to the usual sparsity inducing and fidelity terms, a new term which forces similarity between overlapping patches in the superimposed regions. The functional depends on two free regularization parameters: a coefficient multiplying the sparsity-inducing 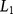 norm of the patch basis functions coefficients, and a coefficient multiplying the 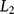 norm of the differences between patches in the overlapping regions. The solution is found by applying the iterative proximal gradient descent method with FISTA acceleration. The gradient is computed by calculating projection of the solution and its error backprojection at each iterative step. We study the quality of the solution, as a function of the regularization parameters and noise, on synthetic data for which the solution is a-priori known. We apply the method on experimental data in the case of Differential Phase Tomography. For this case we use an original approach which consists in using vectorial patches, each patch having two components: one per each gradient component. The resulting algorithm, implemented in the European Synchrotron Radiation Facility tomography reconstruction code PyHST, has proven to be efficient and well-adapted to strongly reduce the required dose and the number of projections in medical tomography.

## Introduction

During the past two decades X-ray Phase Contrast Imaging (PCI) has shown a remarkable enhancement of image contrast and sensitivity for soft tissue. Reducing the deposited dose during PCI-CT is a crucial step towards an eventual clinical implementation of the technique.

A solution to this problem consists in applying iterative CT reconstruction schemes with a-priori knowledge of the solution. The signals occurring in Nature, when cleaned from noise, present most of the time an intrinsic sparsity when expressed in the proper basis. An image is intrinsically sparse when it can be approximated as a linear combination of a small number 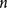 of basis functions, with 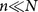, where 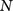 is the image dimensionality. Piece-wise constant images when they are expressed by their gradient are examples of sparse signal: they have non-zero signal only at the borders of flat regions. For piece-wise constant images one can apply very efficient methods based on minimization of a convex functional, called also convex objective function, which contains a total variation penalty term. For other classes of images, such as medical images, one has to choose different solutions which are adapted to the intrinsic sparsity of the case under study (depending on the specific organ and imaging modality). There are mainly two ways: either the sparsity structure is a-priori known and an appropriate basis of functions can be built from the beginning, or it must be automatically learned from a set with the dictionary learning technique [1]. This method consists in building an over-complete basis of functions, over an 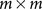 domain, such that, taken an 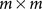 patch from the studied image, the patch can be approximated with good precision as a linear combination of a small number 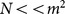 of basis functions. The rationale for using an over-complete basis is that by increasing the basis dimension one increases the number of different patterns that can be fitted using just one or few basis functions. We can think as an example of images containing isolated and weakly curved lines: in this case we could use a basis where each function represents a line with a given intercept and slope, but other functions could be further introduced to fit other shapes. When we fit, patch by patch, a noisy image using the appropriate basis, the features of the original images will be accurately fitted with a small number of components. The noise instead has in general no intrinsic sparsity, and if it happens to have one, it is with high probability very different from the sparsity structure of the original images. Therefore the noise will be reproduced only if we allow a large number of components (the patch basis is over-complete so it can represent the noise) while it will be effectively filtered out if we approximate the noisy image with a small number of components.

The dictionary learning technique has recently been applied to tomography reconstruction using the Orthogonal Matching Pursuit (OMP) denoising procedure [2]. This procedure consists in obtaining first an over-complete basis of functions and then in least-square fitting every patch of the image using at most 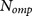 components selected from this basis. The components are heuristically selected choosing, each time, the one having the maximum overlap with the remaining error. This optimization method cannot be implemented as a convex objective function optimization problem, because the linear combination of two candidate solutions can have more than 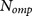 components. In other words, the optimization domain is not convex.

In this paper we present an advanced formalism which implements overlapping patches into a new convex functional that we describe in the section Materials and Methods. For the solution of our functional minimization problem we have applied the recently developed tools taken from the field of convex optimization [3]. Results on both synthetic and experimental data are compared to the state of the art reconstruction methods. We compared the obtained images to Equally Sloped Tomography (EST) [4], TV minimization [5]. Moreover we applied convex optimisation also to another formulation, for the dictionary learning technique, of the objective function, which has been applied, using the non-convex OMP procedure, by Xu et al. [1].

## Methods

### Dictionary Learning

In this section we introduce first the decomposition of an image into non-overlapping patches and the related objective function for denoising. Then we introduce our original formalism which ensures, using overlapping patches, a smooth transition at the patches borders, and finally we apply this formalism to CT reconstruction. In order to make clearer the text we added the following sentences in the introduction of the method: In this approach an iterative loop between the sinogram space and the real space is used. A fidelity term is imposed in the sinogram space while a sparsity inducing term is introduced in the real space.

We denote by 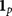 the indicator function of patch p, which is equal to 1 over the patch support (typically an m*m square) and is zero otherwhere. For non-overlapping patches, covering the whole domain, we have:
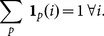
(1)where 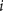 denotes the pixel position and can be thought as a two-dimensional vector. We are looking for the ideal solution 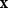 that we express by the vector, 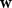, of its coefficients in the basis of patch functions:
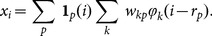
(2)where the set 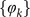 is an over-complete basis of functions over the patch support; 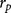 is the closest to the origin corner of the patch 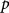, and 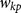 is the component 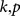 of vector 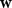 which multiplies the basis function 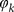 in the patch 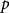. The denoising problem, given an image 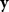, consists in finding the minimum of a functional 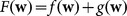 which is sum of two terms. The term 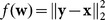 links the solution to the the data 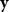. The other term, 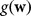, contains the a-priori knowledge about the solution. This way of breaking the functional in two terms has his roots in the Bayesian theorem. From a probabilistic point of view the denoising problem consists in finding, given a noisy image 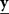 of an object, the most probable object 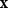 that can generate that image. We represent the object 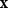 through the patches coefficients 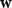. The Bayes theorem, applied to denoising, states that the conditional probability of 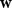 being the exact object given a measurement 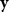, is the product of the probability of 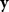 being the measure given the exact solution 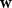 times the a-priori probability of 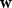.

Assuming gaussian noise, the conditional probability of 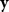 being the measure given the exact solution 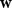 is 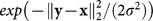, where 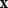 is expressed through the patches coefficients 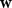 by equation 2. The exact a-priori probability of 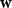 is unknown but we approximate it as 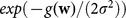. This function expresses our a-priori knowledge that a non sparse solution having a high value of the 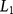 penalization term 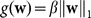 (which is a sparsity-inducing term [6]) has low probability. The most probable solution 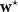 is obtained by finding the 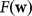 minimum:
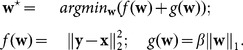
(3)


The solution can be obtained by using the iterative shrinkage thresholding algorithm [3] (ISTA):
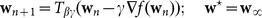
(4)where 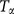 is the shrinkage operator defined as
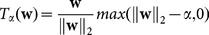
(5)and 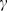 is a positive number lesser than the inverse of the Lipschitz condition number 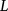:
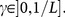
(6)


The Lipschitz number L is such that:
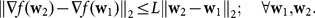
(7)


The ISTA algorithm can be accelerated by the Fast Iterative Shrinkage Thresholding [7](FISTA) method.

In its non-overlapping version, the image denoising with patches is able to detect features that are within the field of the patch: if a line crosses the central region of a patch, it will be detected if the basis of functions has been trained to detect such lines. But in the situation where a line intersects only one point in a corner of the patch square, the signal of this point is indistinguishable from that of a noisy point, no matter the dictionary training.

For this reason the patches denoising technique is often used with overlapping patches using post-process averaging [8]. In this case the minimization problem is solved for each patch separately first, and then the averaging is performed in the overlapped regions.

In this study we do not follow this procedure but we add an overlap term into the objective function. We choose a system of patches which covers the whole domain, and we allow for overlapping. In our implementation of the algorithm the set of all patches is formed by a set of non-overlapping m*m square patches covering the image plus the translated copies that we obtain by 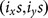 translational vectors, where 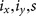 are positive integers, 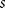 is a constant step size selected by the user and 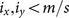.

In the case of overlapping patches the sum of all indicator functions is greater or equal to one:
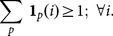
(8)


We define the core indicator functions 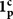, which indicate the core of the patches, and make a non-overlapping covering:
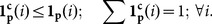
(9)


For our implementation, when the translational step size 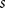 is equal to one, the core region is a pixel at the center of the patch. The core region gets larger when the step size increases.

For a given point 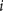, 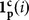 indicates which patch 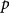 has its center 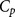 closest to point 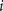:
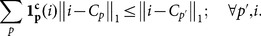
(10)


The solution 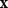 is composed as a function of 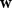 using the central part of the patches as indicated by the functions 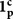:
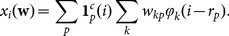
(11)


Now we introduce the 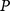 operator which is the projection operator, for tomography reconstruction, and is the identity for image denoising. The functional 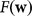 whose minimum gives the optimal solution is written, for both applications, as:
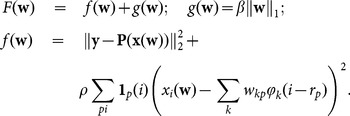
(12)where the 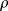 factor weights a similarity-inducing term which pushes all the overlapping patches touching a point 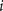, towards the value 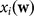 of the global solution 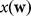 in that point. For future reference we call 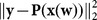 the fidelity term. The factor 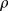 has also the role of a regularisation parameter: as an example we consider the case where the set of overlapping patches is generated with a translational step 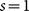. In this case the core indicator function has a 1*1 pixel domain and, without the 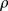 term, we could get a perfect fit, for an arbitrary image, by using for each patch an arbitrary component chosen randomly amongst those which are not zero at the core pixel. The solution is found with the FISTA method, using the gradient of 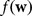 which is easily written as:
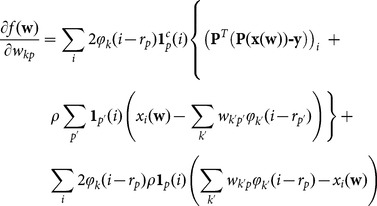
(13)where 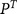 is the adjoint operator of P, called back-projection operator in the case of tomography, which is again the identity for image denoising.

### Other iterative methods

#### Floating Solution Functional

Xu et al. [1] have recently used an objective function which differs from ours for the fact that their global solution 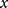 is a free variable, while ours is a function of 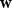: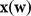. Their objective function is
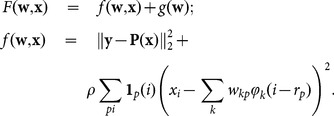
(14)


Xu et al. used the non-convex OMP procedure for minimisation of the functional. In this paper we will also compare their functional to our, using for both functionals the FISTA optimization.

#### Total Variation penalisation

In the total variation (TV) method [5] one minimises a convex functional given by the sum of the fidelity term 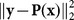 and of a gradient-sparsity inducing term 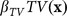, where 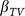 is a regularisation parameter and 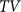 gives the isotropic total variation of the image 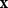: 
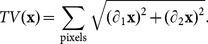
(15)


#### Equally Sloped Tomography

Equally Sloped Tomography (EST) is a Fourier-based iterative reconstruction method that iterates back and forth between real and Fourier space, utilizing an algebraically exact Pseudo-Polar fast Fourier transform [4], [9]. Measured projections (in this case obtained by the radon transform) are inserted into the Fourier space thanks to the fractional Fourier transform (FrFFT). Then the Pseudo-Polar Fast Fourier transform (PPFFT) and its adjoint are utilized to transform the images back and forth between Fourier and object space. During each iteration, physical constraints including sample boundary and the positivity of the coefficient are enforced in real space, while the measured data are applied in the Fourier space. The algorithm, monitored by an error metric, is guided towards the minimum that is consistent with the experimental data. To prevent any human intervention, the algorithm is automatically terminated when no further improvement can be made. In this case the number of iterations was 50 for a good convergence of the algorithm using 80 projections.

The EST algorithm has proved to allow a drastic reduction of the number of projections for conventional CT [9] and phase contrast imaging [4]. Fahimian et al. [9] demonstrated that the image quality and contrast obtained with EST is comparable with other iterative reconstruction schemes such as TV minimization or expected minimization statistical reconstruction with a faster convergence.

## Results

### Numerical Experiment

According to the Shannon-Nyquist criterion, to achieve a proper reconstruction in conventional CT, the number of angular projections 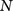 required is determined by 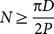
where 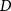 is the thickness of the sample and 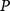 the detector pixel size. One scenario for reducing the deposited dose during a CT scan is to reduce the number of projections.

**Figure 1:**
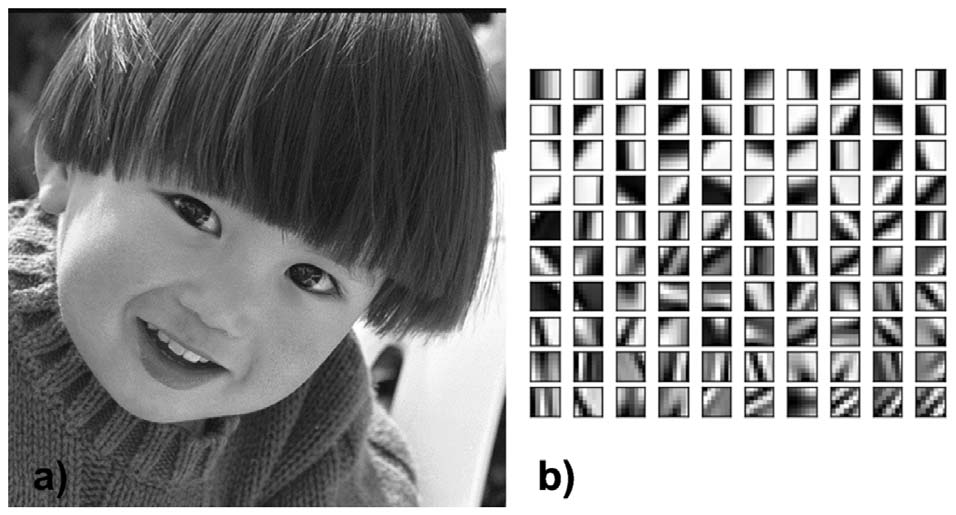
The training image used in our work (a) and the dictionary elements obtained by using the K-SVD algorithm (b). This basis is then used for the numerical experiment.

To investigate the potential of the Dictionary Learning method on synthetic data, we use the standard 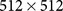 pixel Lena image as phantom. The dictionary is learnt from a different image that the one to reconstruct. In this case we used the boy image showed in fig. 1 a). The dictionary is shown in fig. 1b. Note that we intentionally did not use the standard Shepp-Logan phantom in this study as it is a piece-wise constant and therefore it does not reflect the complexity of a phase contrast medical image.

The sinogram is obtained by projecting the image at 80 angles between 0 and 180 degrees using the radon transform.

**Figure 2:**
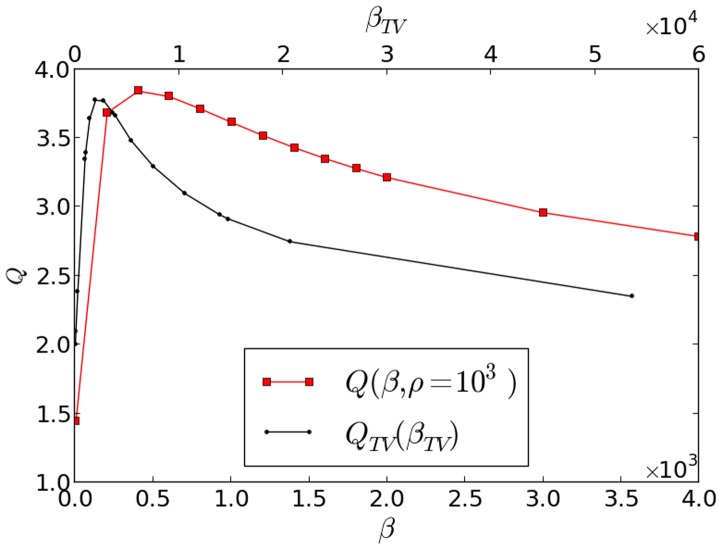
The quality improvement factor 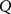 versus the regularisation parameter 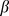 (squares) for our overlapping patches method, and versus 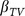 (dots) for the total variation method. The used phantom is the 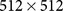 image of Lena, the reconstruction is performed using only 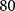 noised projections. For our method we have used a 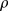 value fixed to 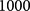, the patches basis shown in figure 1 and a step size of 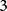.

We have optimized the regularisation parameters maximizing the following improvement factor, which quantifies the improvement obtained with the 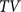 or 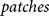 methods, with respect to a simple FBP reconstruction: 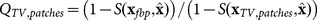
(16)where 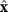 is the exact solution (unnoised Lena) and 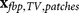 represents the reconstruction result from one of the three methods, and where 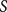 is the Structural SIMilarity index [10] which is equal to 1 when the images are identical. In fig. 2 we show the quality factor dependency versus the regularisation parameter 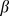 for our overlapping patches method (squares), and versus 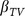
(dots) for the total variation method. For our method we have used a similarity-inducing term (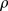) value fixed to 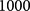, the patches basis shown in fig. 1 and a step size of 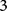.

**Figure 3:**
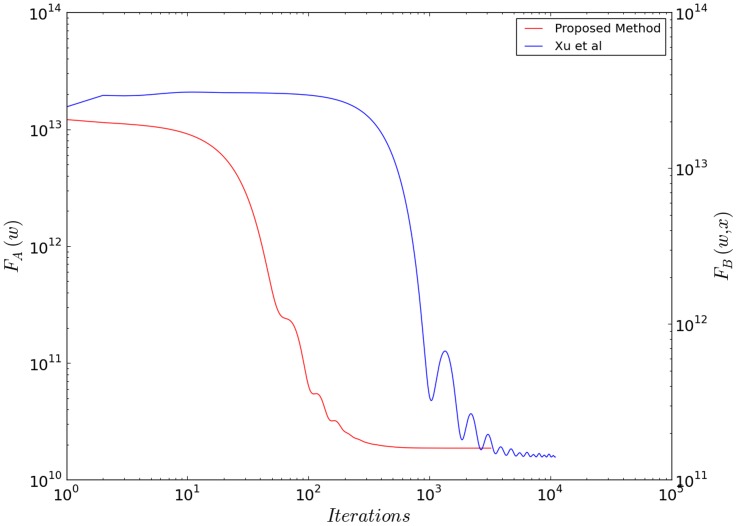
The objective functions 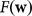 and 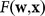 (floating solution form) versus iteration number for FISTA optimisation.

We have performed this reconstruction also with the floating solution function. We have used the same optimisation method, FISTA, used for our functional. We optimized 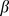 and 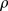 values by scanning over a 2D grid and comparing to the ground-truth. If the ground truth is not available, statistical methods such as the discrepancy principle [11] or generalized cross-validation [12] can be used to select the optimal regularization parameter in future application of the method. We obtained no significant difference between the results obtained with our functional and the floating solution: the SSIM is the same up to the third significant digit, and no significant difference can be detected in the final images. The convergence rate of FISTA, shown for both functional (our method (blue) and [1] (red)) in fig. 3 is instead much faster using our functional form.

**Figure 4:**
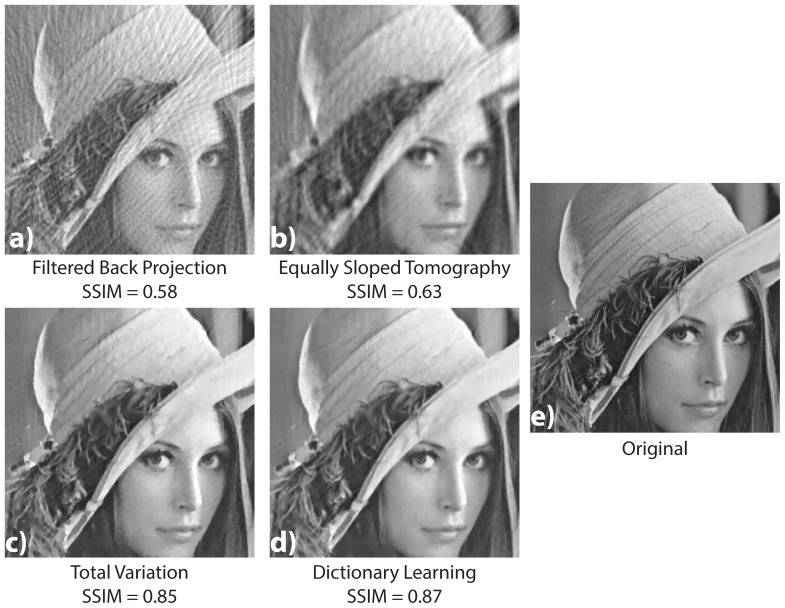
Results from the numerical experiment on the Lena image 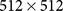. The sinogram is obtained by projecting the image at 80 angles between 0 and 180 degree. A comparison of the images reconstructed with the FBP (a), the EST (b), the 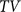 algorithm (c), and our DL method (d) is shown. The final image (e) is the same cropped zone in the original image for the sake of comparison (e).

The comparison of the results obtained by the different reconstruction algorithms is shown in fig. 4. We present the reconstruction results with four methods: filtered backprojection a), EST [9] b), the total variation penalisation [5] c) and our overlapping patches method d). These results have to be compared to the original cropped image shown in subfigure e). In this figure it is clear that the FBP images suffer a lot of streaking artefacts due to the number of projections well below the one required by the Shannon-Nyquist sampling theorem. The EST reconstruction removes some of these artefacts with the price of a blurred image with weak spatial resolution. Despite the fact that our technique gives a 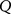 factor which is only slightly better than the one from the 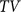 method, the obtained result looks much better by human eye inspection. The 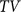 result shows a strong and irregular skin tessellation of those regions which have an illumination gradient. The hat feathers region (second row) is better resolved with the DL method. They look natural in the 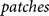 result, while the 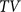 result produces strong grey levels distorsions which vary irregularly along the feathers. EST results show a really good preservation of the tiny structure of the feathers but is noisier. The hat itself looks well preserved in the 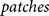 result while, in the 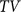 image, the hat borders have irregular shapes. The SSIM values with the original images are reported for each subfigure. These values confirm the observation. Note that the visual difference between TV and DL seems greater than their SSIM values.

1000 iterations were used for the DL method. The computation time is 27 s on a Tesla k20m gnu card using the open source PyHST code [13]. On the same GPU card the computation time for the FBP is less than 1 s and 27 s for the TV method using the same number of iterations. The EST method has not yet been implemented on GPU and is therefore much slower (5 min).

**Figure 5:**
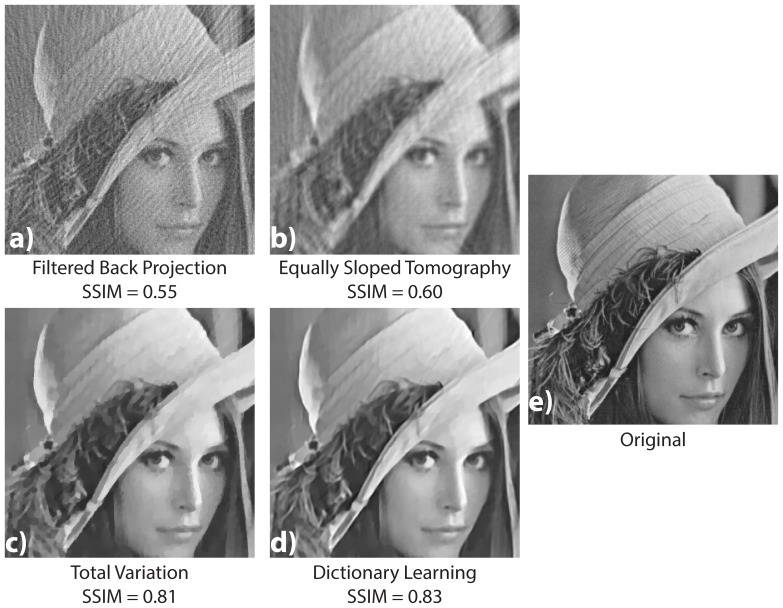
Results from the numerical experiment on the Lena image 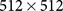 with additive Poisson noise. The sinogram is obtained by projecting the image at 80 angles between 0 and 180 degree, and adding a Poisson noise with a standard deviation equal to 0.3% of the maximal sinogram value. A comparison of the images reconstructed with the FBP (a), the EST (b), the 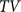 algorithm (c), and our DL method (d) is shown. The final image (e) is the same cropped zone in the original image for the sake of comparison (e).

Another strategy to further reduce the dose in tomography one can acquire with fewer number of photons onto the detector. To simulate this lack of photons we added a Poisson noise onto the sinogram data with a standard deviation 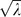 equal to 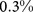 of the sinogram value. We show in fig. 5 the reconstruction results for the different algorithms.

The same conclusions for the not noisy case test can be drawn for the additive Poisson Noise: the dictionary learning method gives the best results. The FBP method is more sensitive to additive noise. With additive noise, the TV method shows a stronger and irregular skin tessellation of the skin surface of Lena.

### Phase Contrast Tomography

In this section we apply our method to medical tomography of a human sample imaged using X-ray Phase Contrat Imaging (PCI).

PCI has shown an enhancement of soft tissue visualization in comparison to conventional imaging modalities [14]. It employs the dual property of X-rays of being simultaneously absorbed and refracted while passing through tissue. Among all the phase contrast techniques, we chose to test our method on analyzer based PCI [15], [16] because of the high sensitivity of the modality. Moreover, to the best of our knowledge, it is the only modality that showed results for investigating large and highly absorbing biological tissues (i.e. full human breasts) at a clinically compatible dose [4].

In the analyzer based PCI technique, the projection data contain a signal which is proportional to the gradient of the X-ray phase in one direction (i.e. the direction perpendicular to the plane formed by the incoming and diffracted X-rays on a perfect Bragg crystal which is used for analyzing the radiation passing through the sample). More details on the principles and technical aspects of PCI are available in [14]. Briefly the analyzer based imaging approach produces a mixed signal which originates from both X-ray absorption and refraction (i.e. phase derivative) [17]. The signal recorded by the detector is therefore very close to those recorded with other PCI techniques such as Grating Interferometry (GI) [18]or Edge Illumination (EI) [19]. All these methods are differential PCI methods and produce similar signals. Therefore the proposed approach can in principle be generalized.

When the object is rotated around an axis (Z-axis, for instance), this signal contains contributions from the X and Y gradient components, where X and Y axes co-rotate with the sample. The two components are de-phased by a rotation angle of 90 degrees and can be reconstructed separately by multiplying before-hand the sinogram with the cosine and sine of the rotation angle. We apply our formalism considering that the reconstructed and learning images are vectorial objects: the value associated with a pixel is not a scalar but a two-component vector.

The studied sample is a 7 cm human breast imaged with a pixel size of 100 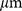. The experiment was conducted at the biomedical beam line of the European Synchrotron Radiation Facility (ESRF). The sample was a human breast mastectomy specimen. The study was performed in accordance with the Declaration of Helsinki. A monochromatic X-ray beam with energy of 60 keV was used.

The training set is obtained from another breast sample imaged with the same technique but with high quality reconstruction. We consider a slice image for which the phase retrieval has been performed [20]. Then we apply a Sobel filter to extract the two derivative components and use the KSVD algorithm [21]. In this experiment 5 iterations were used to obtain the 100 atoms

**Figure 6:**
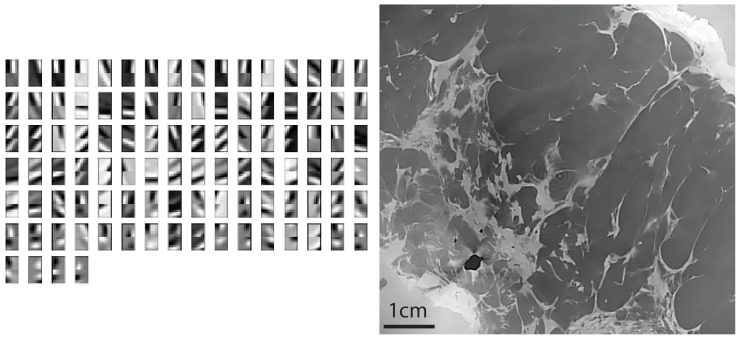
The vectorial basis of patches (left) learnt from a high quality tomographic reconstruction of the phase image of a human breast(right). In each atom of the dictionary the upper 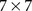 part is the 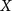 component while the lower part is the 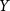 component.

Fig. 6 shows the patches basis functions that we use to fit both components at the same time. The patches size is 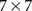 pixels and each basis function is displayed as a 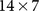 rectangle whose upper 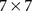 part is the 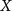 component and the lower one in the 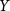 component.

In a previous work [20], it was demonstrated that the CT reconstruction of the refractive index obtained by first reconstructing the CT gradient field images and then applying a phase retrieval procedure, yields a better image quality than performing phase retrieval first and then reconstruction. The method is more robust with respect to noise, which may be a critical aspect in low dose tomography. In this case, the noise level may be such as it covers the information in the region where one gradient component of the refractive index has values close to zero. On the contrary, in those same regions the other component of the gradient of the refractive index has high values and it is thus less sensitive to noise. As a result, the information which is lost in one direction may be somehow retrieved by using the signal contained in the gradient image corresponding to the perpendicular direction. Additionally, when we use the vectorial approach, the information for the two reconstructed gradient components are intrinsically correlated by the dictionary and thus it increases the robustness of the method.

**Figure 7:**
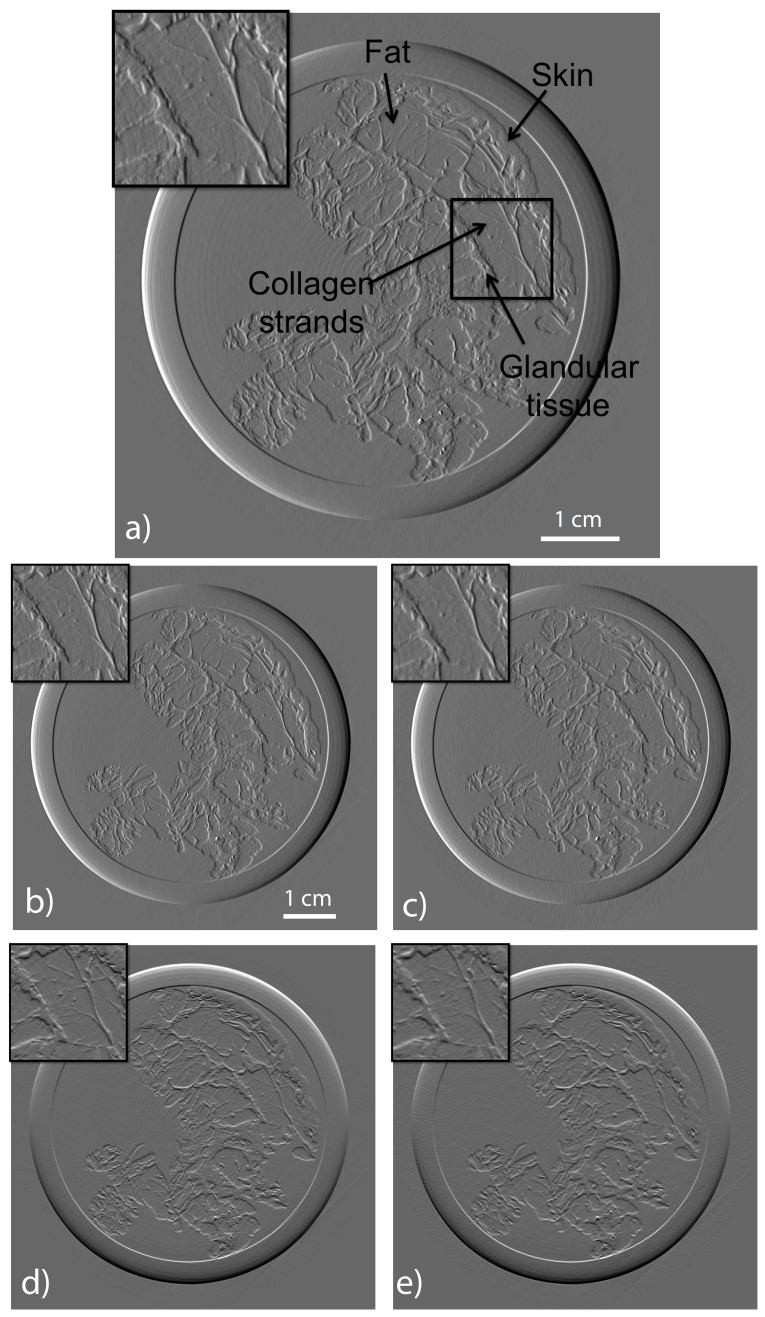
Reconstruction of a computed tomographic slice of the breast. The images on the first and second row are the X and Y phase gradients, respectively. In the left column the results of the reconstruction obtained with the FBP method using the full set of data are reported. In the right column the results of our method using one projection over five are shown. For these reconstructions we set 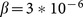 and 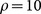.

The result of reconstruction obtained by using filtered back projection algorithm with 1000 projections is shown in fig. 7a. In this image, radiologists could easily identify the skin, fat and glandular tissue. Fig. 7 is the reconstruction of a 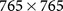 pixel slice, using only 200 projections over the 1000 available. The upper left square is a zoom in the region marked in subfigure 7. The used projections cover, with constant spacing, a 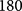 degree range. The right column is the reconstruction with our method for X and Y components, while the left column (subfigure 7c) and d) is reconstructed with the standard FBP using all 1000 available projections. Using our method, we can still generate a high quality image with only one fifth of projections which would otherwise be necessary to generate a high quality reconstruction with the standard FBP method. Visually, the difference between the FBP results obtained with the full data set and our method with a five-fold reduction of the data is barely noticeable. The different borders of structures like skin layers, fatty tissues, and collagen strands are easily identified. The obtained results are very promising and a systematic evaluation for clinical application is under-way. The radiation dose absorbed by the sample during 200 projections is comparable to that of a standard clinical dual view (2D) mammography (3.5 mGy).

**Figure 8:**
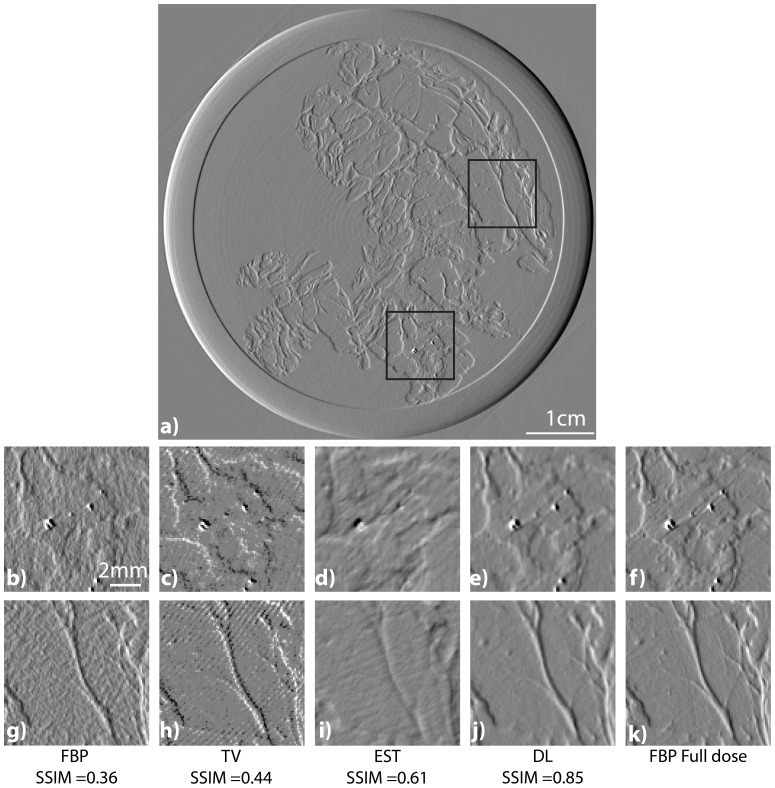
Comparison of the tomographic image reconstructions of the breast obtained with the FBP (b and g), the TV minimization (c and h), the EST (d and i), and our method (e and j) using 200 projections over the 1000 available. The right images (f and k) are the images computed with the FBP using the entire set of projections. The top image (a) is the result obtained by the FBP using the entire set of projections. It is reported for showing the location of the insets. The SSIM values are reported based on the FBP full dose image.

For the sake of comparison we report in fig. 8 the reconstruction obtained using the same number of projections using FBP (subfigure b,g), EST(subfigure d,i) and our method(subfigure f and k) using 200 projections in comparison with the full dose image (subfigure f and k). We report also the results obtaining penalizing the 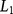 of the reconstructed result(subfigure c and h). Our signal is a derivative, therefore penalizing the derivative modulus is similar to applying the 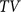 method to the non derived object. The top inset is in a zone close to the skin with a blood vessel. The bottom insets are zoom in a zone with micro-calcifications. Note that micro-calcifications are of high interest for medical diagnoses because it may help identifying malignant masses. We report the ssim values obtained by comparing the images with the FBP reconstruction with the full set of projections

In this figure it is clear that the overall image quality of the TV minimization is poor as well as the FBP with 200 images. The image quality of the EST 200 is lower than DL especially in terms of spatial resolution and sharpness. The EST image is indeed more blurred and the DL looks more similar the original full dose image. The DL image is indeed sharper with a clear delineation of the small micro-calcifications or blood vessel. The EST reconstructed image does not show the little micro-calcification in the middle of the image. Moreover on the top inset small round structures disappeared with the EST reconstruction whilst they are preserved in the DL image. The SSIM values confirm the visual inspection.

## Conclusion

For a decade Iterative CT reconstruction algorithms have demonstrated a possible dose reduction in conventional CT data. To the best of our knowledge, few works dealt with applying those algorithms to phase contrast tomography [4], [22], [23].

We have presented a new convex functional which implements in a mathematically pure form the concept of overlapping-patches-averaging, which was used so-far with a non-convex formalism. The resulting algorithm is efficient and well adapted to strongly reduce the noise in a natural image. A comparison with other iterative algorithms has been carried out on the Lena image showing that our method outperforms TV minimization and Equally Sloped Tomography. The method gives the best results with few projections and is less sensitive to additional noise. Compared to the state of the art dictionary learning method [1] our proposed approach converges faster to an equivalent image quality.

The method was applied to a medical diagnostic case by considering phase contrast tomographic data of a whole cancer-bearing human breast acquired with phase contrast imaging. A vectorial approach consisting of reconstructing gradients of the index of refraction was adopted. We demonstrated that thanks to this approach it is possible to reduce the deposited dose in breast CT by a factor of 5 compared to the standard filtered backprojection while keeping a comparable image quality.

Although we used this specific example as a proof of principle in this study, the method we developed and described can be easily applied to other tomography fields where a limited dose or a rapid acquisition time is a requirement. The numerical results have been generated with PyHST [13], [24], the ESRF tomography reconstruction code which uses the GPU implementation of the presented methods.

### Ethics Statement

The study was performed in accordance with the Declaration of Helsinki. IRB-approval was granted by the ethics committee of the Ludwig-Maximilians University. Written informed consent was gathered before enrollment within the study
